# Serum free 25(OH)D concentrations and cardiovascular disease, heart failure, kidney function decline, and fracture: the health, aging, and body composition study

**DOI:** 10.1093/jbmrpl/ziaf001

**Published:** 2025-01-14

**Authors:** Jonathan H Cheng, Andrew N Hoofnagle, Ronit Katz, Stephen B Kritchevsky, Michael G Shlipak, Mark J Sarnak, Joachim H Ix, Charles Ginsberg

**Affiliations:** Division of Nephrology-Hypertension, University of California, San Diego, CA 92103, United States; Nephrology Section, Veterans Affairs San Diego Healthcare System, San Diego, CA 92161, United States; Departments of Laboratory Medicine and Medicine and the Kidney Research Institute, University of Washington, Seattle, WA 98104, United States; Department of Obstetrics and Gynecology, University of Washington, Seattle, WA 98104, United States; Department of Internal Medicine, Section on Gerontology and Geriatric Medicine, Wake Forest School of Medicine, Winston-Salem, NC 27101, United States; Kidney Health Research Collaborative, Veterans Affairs Medical Center, San Francisco, CA 94121, United States; Kidney Health Research Collaborative, University of California, San Francisco, CA 94121, United States; Division of Nephrology, Department of Medicine, Tufts Medical Center, Boston, MA 02111, United States; Division of Nephrology-Hypertension, University of California, San Diego, CA 92103, United States; Nephrology Section, Veterans Affairs San Diego Healthcare System, San Diego, CA 92161, United States; Division of Nephrology-Hypertension, University of California, San Diego, CA 92103, United States

**Keywords:** vitamin D, heart failure, kidney disease, fracture, cardiovascular disease

## Abstract

Vitamin D deficiency is common across the world. However, the standard clinical biomarker for vitamin D, 25OHD, may be a poor marker of vitamin D status, as most of circulating vitamin D is protein bound and not bioavailable. Free (unbound) vitamin D may therefore be a better marker of vitamin D status. We evaluated the relationship of free vitamin D with incident cardiovascular disease (CVD), heart failure (HF), kidney function decline (KFD) and fracture, among 786 participants in the Health Aging and body composition study. We used sequential models to assess hazard ratios (HRs) of each outcome that adjusted for age, sex, race, season of blood sampling, and study site, kidney function, serum calcium and phosphate, FGF 23, PTH, BMI, and vitamin D supplementation. The mean age of the 786 participants was 75 ± 3 yr, 53% were women, and 40% were Black. The median free vitamin D concentration was 5.3 (interquartile range 4.1-6.7) pg/mL. There were 157 cases of incident CVD, 123 cases of incident HF, 382 cases of incident KFD, and 178 fractures over 11 yr of follow-up. In fully adjusted models, a 2-fold greater free vitamin D was associated with lower risk of incident HF [HR 0.75, 95%CI,0.58-0.96 ] and greater risk KFD [1.25(1.03-1.52)]. We found no association between free vitamin D and incident CVD or fracture. We did not find evidence that free vitamin D was a superior marker of clinical outcomes compared to total 25OHD alone. Further studies are needed to elucidate the relationship of free vitamin D with clinical outcomes.

Vitamin D deficiency, defined as 25(OH)D < 20 ng/mL, is hypothesized to increase the risk of various health problems including mineral bone diseases, such as osteoporosis and rickets, kidney disease, and cardiovascular disease (CVD).^1^^,^^2^ However, 25(OH)D may not be a reliable biomarker in determining important clinical outcomes due to variability in vitamin D-binding protein (VDBP).^3^ 25(OH)D exists as both free (~1%) or protein bound (~99% to VDBP with a small fraction to albumin) and the 25(OH)D assay measures total vitamin D, irrespective of protein binding.^4^ Previous studies have shown substantial variability in VDBP concentrations by over 30% across individuals, and common genetic variants in VDBP may lead to different binding affinity for 25(OH)D.^4–7^ In contrast, free, biologically active vitamin D may not be affected by VDBP concentrations or phenotypes. In this study, we evaluated the relationship of free vitamin D with bone, kidney, and heart outcomes in healthy, community-dwelling older adults who participated in the Health, Aging, and Body Composition (Health ABC) Study.^8^ We hypothesized that a lower free vitamin D concentration would be associated with worse clinical outcomes including increased risk of incident CVD, heart failure (HF), kidney function decline (KFD) (≥30% decline in cystatin-based estimated glomerular filtration rate), and fracture.

The Health ABC study is an, longitudinal study designed to evaluate risk factors for the functional decline in older persons. Free 25(OH)D was quantified using the Diasource competitive ELISA with an intra-assay coefficient of variation of the assay was 8.9% to 16.7%. Total 25(OH)D was performed using liquid chromatography with tandem mass spectrometry (LC–MS/MS) with a CV of <5.6%. These vitamin D measures were performed in 786 participants (459 of which were part of a randomly selected subcohort) as part of an ancillary study evaluating calcitriol and kidney disease, CVD, and all-cause mortality.^9^ We used a case-cohort design to evaluate the associations of free vitamin D and total 25(OH)D with incident CVD (157 total cases, 89 within the subcohort), incident HF (123 total cases, 53 within the subcohort), KFD (382 total cases, 74 within the subcohort), and fractures (178 total cases, 97 within the subcohort). Methodology of adjudication of the events has been defined previously.^9^ Participants in the subcohort were given weights based on the inverse probability of their selection, while cases occurring outside the subcohort were not assigned weights until the time of failure. We used sequential models to assess hazard ratios (HRs) that adjusted for age, sex, race, season of blood sampling, and study site, estimated flomerular filtration rate (eGFR) (CKD-EPI cystatin C equation), serum calcium and phosphate, FGF 23, PTH, BMI, and vitamin D supplementation.^10^ Variables that were not normally distributed were log transformed.

The mean age of the 786 participants was 75 ± 3 yr, 53% were women, 40% were Black, and the mean eGFR was 67 ± 16 mL/min/1.73m^2^. The median free vitamin D concentration was 5.3 (interquartile range 4.1-6.7) pg/mL, and the median total 25(OH)D was 23.3(15.2, 31.5) ng/mL. Further baseline characteristics of this cohort have been described previously and are in Table S1.^11^ Notably, compared to persons in the lowest free 25(OH)D quartile, those with higher free 25(OH)D concentrations were more often male, white, and had lower BMIs. Persons with higher free 25(OH)D also had lower PTH and higher FGF 23 concentrations. In fully adjusted models, a 2-fold greater free vitamin D was associated with lower risk of incident HF [HR 0.75, 95%CI, 0.58-0.96] and greater risk KFD [1.25(1.03-1.52)] **(**Table 1**)**. Free vitamin D was not significantly associated with incident CVD [0.87(0.66, 1.16)] or fracture [1.00(0.66-1.50)]. A 2-fold greater total 25(OH)D was associated with a lower risk of incident HF[0.71(0.56-0.90)]. Total 25(OH)D was not associated with KFD [1.12 (0.96-1.30)], incident CVD [0.82 (0.63-1.05)], or fracture [0.92 (0.66-1.27)]. In post hoc analyses, we evaluated if associations were modified by sex and BMI, as vitamin D is known to have varying effects on muscle and in persons who are overweight. We found that the relationships of free vitamin D with incident HF [HR 0.66, (0.46, 0.97)] and major KFD [1.33 (1.02, 1.75)] as well as the relationship of total 25(OH)D with incident HF [0.76 (0.59, 0.97)] were primarily observed among women. In analyses stratified by BMI ≥ 25 kg/m^2^, we found that the association of free vitamin D with incident HF was primarily in persons who were overweight [0.67 (0.49, 0.92)]. Finally, the relationship of total 25(OH)D with incident CVD and HF was primarily in persons who were overweight as well [0.72 (0.54, 0.97), and 0.77 (0.62, 0.97), respectively].

**Table 1:** Association of vitamin D metabolites and clinical outcomes among 786 health aging and body composition participants.^a^

	*N* Total events (subcohort)	Mean time until event (yr)	Free 25(OH)D		Total 25(OH)D	
			HR per 100% higher (95%CI)	*p*	HR per 100% higher (95%CI)	*p*
**Incident CVD**	157 (89)	10.3				
** Model 1**			0.92 (0.71-1.19)	.63	0.80 0.63, 1.02)	.07
** Model 2**			0.87 (0.66-1.16)	.52	0.82 (0.63, 1.05)	.12
**Incident HF**	123 (56)	8.2				
** Model 1**			0.79 (0.62-1.01)	.06	0.74 (0.58, 0.94)	.02
** Model 2**			0.75 (0.58-0.96)	.03	0.71 (0.56, 0.90)	.01
**Kidney function decline**	382 (74)	7.3				
** Model 1**			1.19 (0.99-1.42)	.06	1.07 (0.93, 1.22)	.36
** Model 2**			1.25 (1.03-1.52)	.02	1.12 (0.96, 1.30)	.16
**Fracture**	178 (97)	10.2				
** Model 1**			1.00 (0.67-1.47)	.98	0.93 (0.70, 1.23)	.63
** Model 2**			1.00 (0.66-1.50)	.99	0.92 (0.66, 1.27)	.60

Model 1 adjusted for age, sex, race, season of measurements, site of measurement. Model 2 adjusted for the same variables as model 2 as well as eGFR, serum calcium, phosphate, FGF-23, PTH, BMI, vitamin D supplementation.

Abbreviations: CVD, cardiovascular disease; HF, heart failure; HR, hazard ratio.

a 489 participants from random subcohort and remainder selected based on case-cohort design.

Initially, we hypothesized that higher concentration of free vitamin D would be linked with a decreased risk of adverse clinical outcomes among older adults residing in the community. However, our analysis yielded mixed results. We found that a 2-fold higher free vitamin D concentration was associated with a 25% lower risk of developing HF, a 25% higher risk of experiencing significant decline in kidney function, and no correlation with incident CVD or fracture. Multiple studies have shown free vitamin D is associated with a reduction in the risk of HF, possibly due to its anti-inflammatory properties, oxidative stress reduction, and regulation of calcium levels in heart muscle cells.^12^ There are known vitamin D receptors in the human heart, suggesting activated vitamin D (calcitriol) has an effect on cardiac function. However, we are not aware of studies on free or bioavailable vitamin D and cardiovascular outcomes or function. The link between measured free vitamin D and KFD is poorly explored, and prior studies examining the association between 25(OH)D and KFD have generated mixed findings.^9^^,^^13^ One potential explanation for the inverse relationship of 25(OH)D and loss of kidney function, is that elevated free vitamin D levels may increase the risk of vascular calcification, ultimately leading to kidney disease. However, if this mechanism were valid, a higher risk of CVD would be anticipated, which we did not observe. With regard to the lack of relationship of free vitamin D with incident CVD or fracture, free vitamin D appears to have more intra-individual variability than 25(OH)D and therefore a single measurement may not be useful to predict these important clinical outcomes. Further research is required to examine the association between free vitamin D and cardiovascular outcomes.

Strengths of this study include the availability of both free and total vitamin D measurements concurrently, and extensive confounding data including baseline kidney function and other bone and mineral biomarkers. The study population also had a large number of clinically relevant outcomes and had an almost even split of both sexes from multiple clinical sites, increasing generalizability. Limitations of this study include that it is an observational study; therefore, we are unable to make cause-effect relationships. Additionally, the mixed findings of possible benefits for the heart and harm in the kidney make the utility of this biomarker unclear and require caution when interpreting. Finally, considering these mixed relationships, the physiology underlying the effects of vitamin D in the heart and the kidney need to be further elucidated.

In conclusion, in a population of older community-dwelling adults, a 2-fold greater level of free vitamin D was associated with a decreased risk of HF, an increased risk of kidney function decline, and was not associated with incident CVD or fracture. The association of free vitamin D with incident HF was similar to that of total 25(OH)D. Thus, while free vitamin D was associated with certain clinical outcomes, the results were inconsistent. Therefore, free vitamin D may not be appropriate as a clinical biomarker of vitamin D status to replace total 25(OH)D.

## Supplementary Material

Supplemental_Table_1_ziaf001

## Data Availability

The data underlying this article are available in the National Institute on Aging repository, at https://www.nia.nih.gov/healthabc-study.
