# Supplementary material for: Serum free 25(OH)D concentrations and cardiovascular disease, heart failure, kidney function decline, and fracture: the health, aging, and body composition study
Source: JBMR Plus. 2025 Jan 14;9(3):ziaf001. doi: 10.1093/jbmrpl/ziaf001 (PMC11807283; doi:10.1093/jbmrpl/ziaf001)
Supplement: Supplemental_Table_1_ziaf001 [file supplemental_table_1_ziaf001.docx]

| **Supplemental Table 1. Baseline Characteristics of Health ABC Participants by Free Vitamin D Quartile** | | | | |
| --- | --- | --- | --- | --- |
|  | **Quartile 1 (n=197)** | **Quartile 2 (n=196)** | **Quartile 3 (n= 197)** | **Quartile 4 (n=196)** |
| Range (pg/ml) | 0.81-4.08 | 4.10-5.28 | 5.29-6.69 | 6.70-22.79 |
| Age (years) ± SD | 74 (3) | 74 (3) | 75 (3) | 75 (3) |
| Female, n (%) | 127 (65) | 95 (48) | 94 (48) | 65 (33) |
| White, n (%) | 73 (37) | 102 (52) | 127 (65) | 155 (79) |
| Clinic site, n(%) |  |  |  |  |
| Memphis | 100 (51) | 114 (58) | 92 (47) | 98 (50) |
| Pittsburgh | 87 (49) | 82 (42) | 105 (53) | 98 (50) |
| Season of blood measurement, n (%) |  |  |  |  |
| Winter | 45 (23) | 61 (31) | 40 (20) | 49 (25) |
| Spring | 69 (35) | 248(24) | 52 (26) | 31 (16) |
| Summer | 13 (25) | 35 (18) | 55 (28) | 74 (38) |
| Fall | 58 (29) | 52 (27) | 50 (25) | 42 (21) |
| BMI (kg/m^2^) ± SD | 28.7 (5.0) | 27.8 (4.8) | 27.4 (5.0) | 26.1 (4.1) |
| Smoking status, n (%) |  |  |  |  |
| Never | 90 (46) | 84 (43) | 76 (39) | 86 (44) |
| Former | 89 (45) | 94 (48) | 103 (52) | 96 (49) |
| Current | 18 (9) | 18 (9) | 19 (9) | 14 (7) |
| Diabetes, n (%) | 94 (48) | 93 (47) | 77 (39) | 65 (33) |
| Systolic BP (mm Hg) ± SD | 138 (22) | 137 (21) | 130 (19) | 132 (21) |
| On anti-HTN medications, n (%) | 127 (65) | 130 (66) | 124 (63) | 111 (57) |
| eGFR (ml/min/1.73m^2^) ± SD | 72 (21) | 75 (19) | 73 (19) | 72 (19) |
| Albumin/Creatinine, median [IQR] | 8 [4-40] | 9 [5-31] | 7 [4-19] | 7 [4-18] |
| Calcium (mg/dl) ± SD | 8.9 (0.5) | 8.8 (0.4) | 8.9 (0.4) | 8.8 (0.4) |
| Phosphate (mg/dl) ± SD | 3.6 (0.5) | 3.5 (0.5) | 3.6 (0.5) | 3.5 (0.5) |
| PTH (pg/ml), median [IQR] | 43 [30-58] | 34 [26-47] | 31 [24-40] | 29 [22-39] |
| FGF23 (pg/ml), median [IQR] | 43 [31-56] | 42 [32-55] | 45 [35-60] | 48 [38-63] |
| Vitamin D Supplement, n (%) | 9 (5) | 15 (8) | 24 (12) | 21 (11) |
